# Acceptability of HIV self-testing to support pre-exposure prophylaxis among female sex workers in Uganda and Zambia: results from two randomized controlled trials

**DOI:** 10.1186/s12879-018-3415-z

**Published:** 2018-10-04

**Authors:** Katrina F. Ortblad, Michael M. Chanda, Daniel Kibuuka Musoke, Thomson Ngabirano, Magdalene Mwale, Aidah Nakitende, Steven Chongo, Nyambe Kamungoma, Catherine Kanchele, Till Bärnighausen, Catherine E. Oldenburg

**Affiliations:** 1 International Clinical Research Center, University of Washington, Seattle, USA; 2 John Snow, Inc, Lusaka, Zambia; 3 International Research Consortium, Kampala, Uganda; 4 Uganda Health Marketing Group, Kampala, Uganda; 5 Department of Global Health and Population, Harvard T.H. Chan School of Public Health, Boston, USA; 6 Africa Health Research Institute, KwaZulu-Natal, South Africa; 7 Heidelberg Institute of Public Health, University of Heidelberg, Heidelberg, Germany; 8 Francis I. Proctor Foundation, University of California San Francisco, 513 Parnassus Ave, Room S334J, San Francisco, CA USA; 9 Department of Ophthalmology, University of California, San Francisco, San Francisco, USA; 10 Department of Epidemiology & Biostatistics, University of California, San Francisco, USA

**Keywords:** Pre-exposure prophylaxis, Female sex workers, HIV self-testing, Zambia, Uganda

## Abstract

**Background:**

HIV pre-exposure prophylaxis (PrEP) is highly effective for prevention of HIV acquisition, but requires HIV testing at regular intervals. Female sex workers (FSWs) are a priority population for HIV prevention interventions in many settings, but face barriers to accessing healthcare. Here, we assessed the acceptability of HIV self-testing for regular HIV testing during PrEP implementation among FSWs participating in a randomized controlled trial of HIV self-testing delivery models.

**Methods:**

We used data from two HIV self-testing randomized controlled trials with identical protocols in Zambia and in Uganda. From September–October 2016, participants were randomized in groups to: (1) direct delivery of an HIV self-test, (2) delivery of a coupon, exchangeable for an HIV self-test at nearby health clinics, or (3) standard HIV testing services. Participants completed assessments at baseline and 4 weeks. Participants reporting their last HIV test was negative were asked about their interest in various PrEP modalities and their HIV testing preferences. We used mixed effects logistic regression models to measure differences in outcomes across randomization arms at four weeks.

**Results:**

At 4 weeks, 633 participants in Zambia and 749 participants in Uganda reported testing negative at their last HIV test. The majority of participants in both studies were “very interested” in daily oral PrEP (91% Zambia; 66% Uganda) and preferred HIV self-testing to standard testing services while on PrEP (87% Zambia; 82% Uganda). Participants in the HIV self-testing intervention arms more often reported preference for HIV self-testing compared to standard testing services to support PrEP in both Zambia (*P* = 0.002) and Uganda (*P* < 0.001).

**Conclusion:**

PrEP implementation programs for FSW could consider inclusion of HIV self-testing to reduce the clinic-based HIV testing burden.

**Trial registration:**

ClinicalTrials.gov NCT02827240 and NCT02846402.

## Background

HIV pre-exposure prophylaxis (PrEP) is highly efficacious for the prevention of HIV acquisition in men who have sex with men [1] and heterosexual serodiscordant couples [2, 3]. Although few HIV seroconversions have been documented in the context of PrEP implementation programs in the United States [4–7], there is concern related to the development of resistance for individuals who acquire HIV while taking PrEP [7]. Routine HIV testing is therefore a core component of patient management in PrEP programs. The World Health Organization [8] and the Centers for Disease Control and Prevention [9] recommend quarterly HIV testing for all individuals taking PrEP. However, frequent HIV testing can be burdensome to some PrEP patients, particularly for those who face logistical barriers to accessing healthcare [10].

HIV self-testing allows individuals to test for HIV at the time and place of their choosing, and may reduce some barriers to accessing regular HIV testing. In the context of PrEP implementation programs, HIV self-testing might be a useful complement to clinic-based testing [11]. For example, if the sensitivity and specificity of HIV self-testing is adequate for people taking PrEP [12], HIV self-testing could be used to screen for HIV instead of clinic visits for some time points. HIV self-testing has generally been shown to be acceptable [13, 14] and has been implemented in diverse populations of users [15].

Female sex workers (FSW) in Sub-Saharan Africa are disproportionately affected by the HIV epidemic [16]. PrEP has the potential to substantially alter the course of the HIV epidemic among FSW, particularly as a user-controlled prevention intervention that does not require negotiation with a partner, such as male condom use [17]. FSW often receive a higher price for engaging in condomless sex, and thus may be economically disincentivized from using male condoms in particular [18]. PrEP may therefore be a beneficial tool for FSW in some contexts [19]. A demonstration project of PrEP integration into routine care for FSW in South Africa demonstrated good uptake but suboptimal retention in PrEP care [20]. Barriers to accessing routine HIV testing exist for many populations of FSW, including logistical barriers such as timing of clinic hours and interpersonal barriers such as anticipated stigma from healthcare workers [21–24], which may contribute to poor retention in PrEP care.

Here, we report PrEP acceptability among FSW participating in two trials of oral HIV self-testing delivery models in Zambia [25] and Uganda [26], and the acceptability of HIV self-testing in the context of PrEP use.

## Methods

### Participants and procedures

We conducted two separate three-arm cluster randomized trials of HIV self-testing delivery models for FSWs in three Zambian transit towns (ClinicalTrials.gov NCT02827240) [25] and urban Uganda (ClinicalTrials.gov NCT02846402) [26]. The two trials followed identical protocols and measured identical outcomes at each time point [27]. The present analysis is a pre-specified secondary analysis from each parent trial.

Trained peer educators each recruited 6 participants in Zambia and 8 in Uganda. In Zambia, participants were recruited in Livingstone, Kapiri Mposhi, and Chirundu, all transit towns. In Uganda, participants were recruited in Kampala, the capital city. In each country, individuals were eligible for participation if they were at least 18 years of age, reported exchanging sex for money or goods at least once in the previous month, self-reported an HIV negative status and no recent (< 3 months) HIV test or an unknown HIV serostatus, and were permanent residence in their town/city of recruitment.

All participants completed four peer educator visits at weeks 0, 2, 6, and 10, and three study visits at baseline prior to randomization and 4 and 16 weeks after the first peer educator visit. We restricted our analysis to participants who self-reported that the results of their most recent HIV test were negative at the 4-week study visit. PrEP-related outcomes were only measured at the 4-week study visit, and thus no 16-week data were included in this analysis.

### Randomization

Participants were randomized in groups of one peer educator and 6 participants in Zambia and 8 participants in Uganda. Groups were randomized in a 1:1:1 fashion to one of three randomization arms: 1) direct distribution of the HIV self-test kit from the peer educator to the participant (*direct delivery*), 2) distribution of a coupon from the peer educator to the participant that could be used to collect the HIV self-test from an existing health facility (*coupon delivery*), or 3) referral from the peer educator to existing and free of charge standard HIV testing facilities (*standard of care*). The randomization list was generated in R (Version 3.3.1, The R Foundation for Statistical Computing, Vienna, Austria) in random blocks of size 3, 6, and 9. A separate randomization list was generated for the Zambia and Uganda studies. In both countries, the randomized study assignments were placed in opaque envelopes that were opened by a peer educator and a study staff member once each peer educator group enrolled its target number of participants.

### Interventions

All participants completed four peer educator visits, at weeks 0, 2, 6, and 10. At baseline, the peer educator visit was done in a group setting and consisted of HIV prevention counseling, distribution of male condoms, and discussion of where participants could go for HIV testing. In the HIV self-testing arms, participants were additionally given either an HIV self-test kit (direct delivery arm) or coupon that could be used for collection of an HIV self-test at a participating pharmacy or health clinic (coupon delivery). We used the OraQuick Rapid HIV-1/2 Antibody Test (OraSure Technologies, Bethlehem, PA), which has been shown to have a sensitivity of 98.7% and specificity of 99.8% in Zambia under field conditions [28]. Subsequent peer educator visits were one-on-one, and consisted of screening for adverse events, distribution of male condoms, and additional discussion related to HIV testing. Participants in the HIV self-testing arms were also able to ask their peer educator if they needed any help with HIV self-testing. At the fourth peer educator visit (week 10), participants in the HIV self-testing arms received a second HIV self-test or coupon for collection of an HIV self-test at a health clinic.

### Measures

Participants completed three surveys using computer-assisted personal interview with a trained research assistant. Interviews occurred at baseline prior to randomization and weeks 4 and 16 following the first peer educator visit.

#### Demographic characteristics

At baseline, participants were asked their age, literacy (if they could read and write), if they owned a mobile phone, monthly income (Kwacha in Zambia or Ugandan Shillings in Uganda), and if they had a primary partner (e.g., husband or boyfriend who is not a client). Participants were also asked the age at which they began working in sex work. The number of years participants engaged in sex work was calculated as the difference in their current age and the age at which they reported starting working in sex work. Participants were additionally asked to report the number of clients they had on an average working night, and how many of these they used a condom (male or female) with on average. Participants were categorized as inconsistently using condoms (male or female) with clients if the number of clients with whom they used a condom (male or female) on an average night was less than their average number of clients per night. Finally, participants were asked to estimate how likely it was, on a 10-point ladder scale, that they would acquire HIV in the next year.

#### PrEP acceptability

At the 4-week visit, research assistants read a brief script to participants that introduced daily oral PrEP. The script described daily oral PrEP as a new method for preventing HIV for people who do not have HIV but are at risk of getting it. Participants were told by the research assistant that PrEP has been shown to reduce the risk of HIV infection when taken consistently. Participants were asked how interested they were in taking daily oral PrEP, and their responses were categorized either as “very interested” or less. Injectable PrEP, PrEP in the form of a vaginal microbicide, and a vaginal ring were then introduced to participants as forms of PrEP that were being studied. Injectable PrEP was described as a shot that is given every 3 months. Vaginal microbicides were described as gels or lubricants that were inserted into the participant’s vagina. Vaginal rings were described as rings that are inserted into the vagina that lasts for one month. Participants were asked how interested they were in taking each PrEP modality, interest was again categorized as “very interested” or less.

#### Acceptability of HIV self-testing during PrEP use

Participants were told by the research assistant that individuals taking PrEP should be tested for HIV every 3 months. Participants were then asked if they would be willing to test for HIV every three months. This question did not specify which type of testing (e.g., self-test or standard facility-based testing). Finally, participants were asked if they would prefer standard HIV testing at a clinic or HIV self-testing for testing while taking PrEP.

### Statistical analysis

Descriptive characteristics were calculated with proportions for categorical variables and medians and interquartile ranges (IQR) for continuous variables. We calculated the proportion of participants who responded that they would be “very willing” to use each PrEP modality across all study arms. To determine whether exposure to the HIV self-testing intervention led to increased willingness to test for HIV as part of a PrEP program or preference for HIV self-testing versus standard testing, we used mixed effects logistic regression model with a fixed effect for study arm and a random effect for peer educator group. All analyses were conducted separately for the Uganda and Zambia studies. All *P*-values were two-sided and analyses were run in Stata 14.1 (StataCorp, College Station, TX).

## Results

**Fig. 1:**
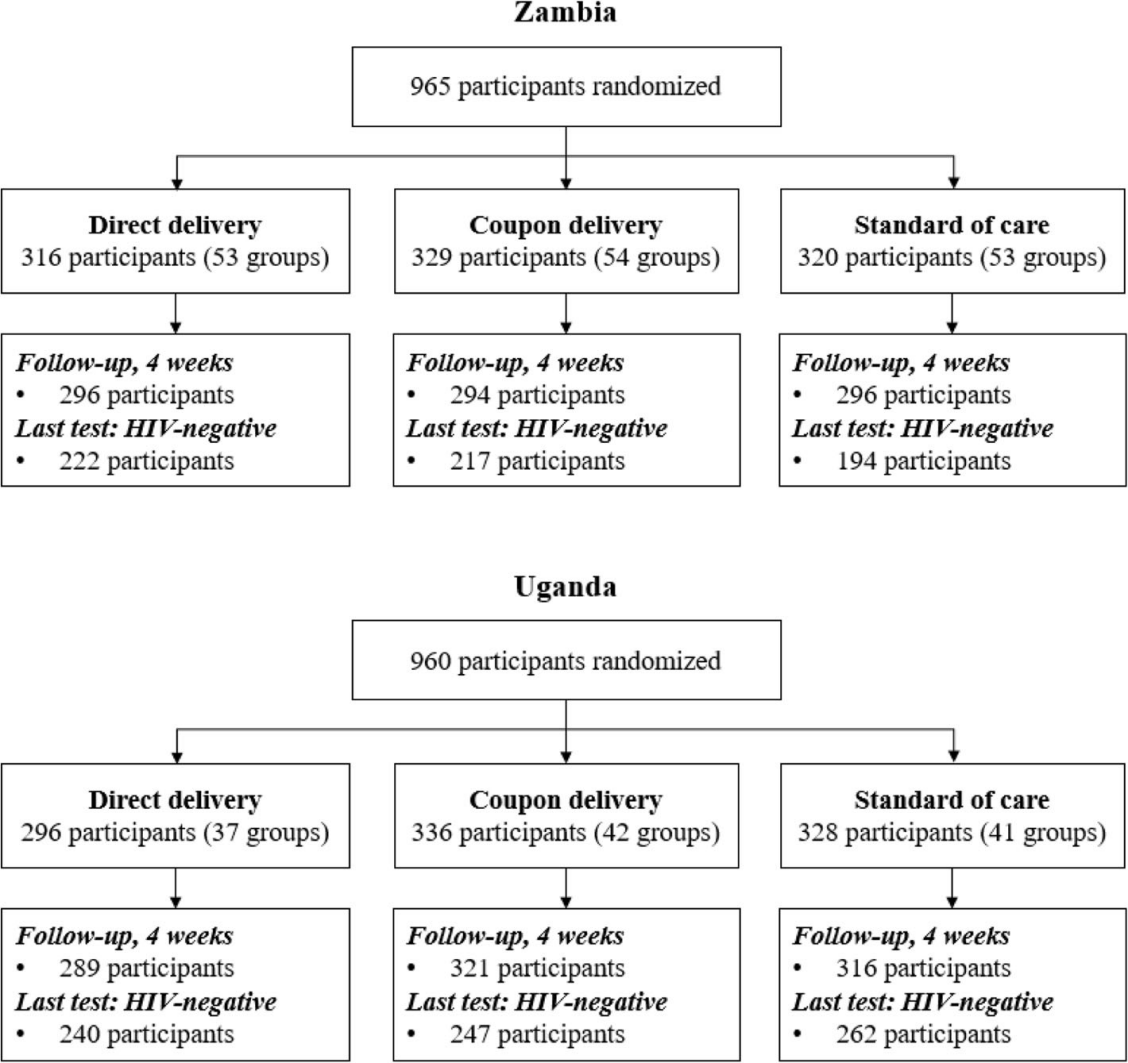
Flow of participants included in the study in both Zambia and Uganda

From September–October of 2016, 965 participants enrolled in the Zambia trial and 960 participants enrolled in the Uganda trial (Fig. 1). At the four-week study visit, retention of study participants in the Zambia trial was 92% (886/965) and retention of study participants in the Uganda trial was 96% (926/960). Of these participants, 71% (633/886) in Zambia and 81% (749/926) in Uganda reported testing HIV-negative at their last HIV test and were included in our study population.

Table 1 shows the demographic characteristics of participants in our study populations at the baseline visit. Participants in Zambia were slightly younger than participants in Uganda. In Zambia, the mean age of study participants was 24 years (interquartile range [IQR] 21 to 29), while in Uganda the median age of study participants was 28 years (IQR 24 to 32). The majority of participants in both studies had a primary sexual partner, self-reported the ability to read and write and owned a mobile phone. Compared to participants in Uganda, those in Zambia tended to be newer to sex work, had a higher prevalence of inconsistent condom use with clients, and perceived themselves at greater risk of acquiring HIV in the next year. There were no statistically significant differences in demographic characteristics at baseline across the three randomization arms in both Zambia and Uganda.

**Table 1:** Baseline demographic characteristics of the study sample

	Direct HIVST distribution	HIVST coupon distribution	Standard testing
Zambia			
Sample Size	*N* = 222	*N* = 217	*N* = 194
Age (med, IQR)	24 (20 to 28)	24 (21 to 29)	24 (21 to 29)
Have a primary partner	127 (57%)	133 (61%)	121 (62%)
Can read and write	175 (79%)	176 (81%)	137 (71%)
Mobile phone ownership	185 (83%)	190 (88%)	170 (88%)
Monthly income			
No income	48 (22%)	50 (23%)	45 (%)
< 250 kwacha^a^	21 (10%)	40 (19%)	28 (%)
251–500 kwacha^a^	58 (26%)	41 (19%)	42 (%)
501–1000 kwacha^a^	55 (25%)	57 (27%)	44 (%)
1001–1500 kwacha^a^	21 (10%)	15 (7%)	13 (%)
> 1500 kwacha^a^	17 (8%)	11 (5%)	19 (%)
Years in sex work (med, IQR)	4 (2 to 8)	5 (3 to 8)	3 (5 to 9)
Inconsistent condom use with clients	169 (77%)	152 (71%)	144 (76%)
Risk of acquiring HIV in next year, 10-point scale^b^ (med, IQR)	6 (5 to 8)	6 (5 to 7)	5 (4 to 7)
Uganda			
Sample size	*N* = 247	*N* = 240	*N* = 262
Age (med, IQR)	27.5 (24 to 31)	28 (24 to 31)	28 (24 to 32)
Have a primary partner	160 (67%)	141 (57%)	156 (60%)
Can read and write	209 (87.1)	209 (84.6)	228 (88.0)
Mobile phone ownership	235 (98%)	233 (94%)	249 (95%)
Monthly income			
No income	2 (1%)	0 (0%)	0 (0%)
< 250 UGX^c^	50 (21%)	56 (23%)	41 (16%)
251–500 UGX^c^	69 (29%)	80 (33%)	106 (41%)
501–1000 UGX^c^	88 (37%)	83 (34%)	90 (35%)
1001–1500 UGX^c^	27 (11%)	19 (8%)	22 (8%)
> 1500 UGX^c^	4 (2%)	5 (2%)	2 (1%)
Years in sex work (med, IQR)	5 (3 to 8)	4 (3 to 8)	5 (2 to 8)
Inconsistent condom use with clients	104 (44%)	101 (41%)	104 (40%)
Risk of acquiring HIV in next year, 10-point scale^b^ (med, IQR)	4 (2 to 6)	5 (3 to 6)	4 (2 to 5)

^a^Exchange rate: 1 USD = 9.2 Zambian Kwacha (September 2016)

^b^Participants were asked to indicated how likely it is that they will contract HIV in the next year using a 10-rung ladder scale. Larger scores indicated a greater likelihood of getting HIV

^c^Exchange rate: 1 USD = 3366 Uganda shilling (UGX) (October 2016)

**Fig. 2:**
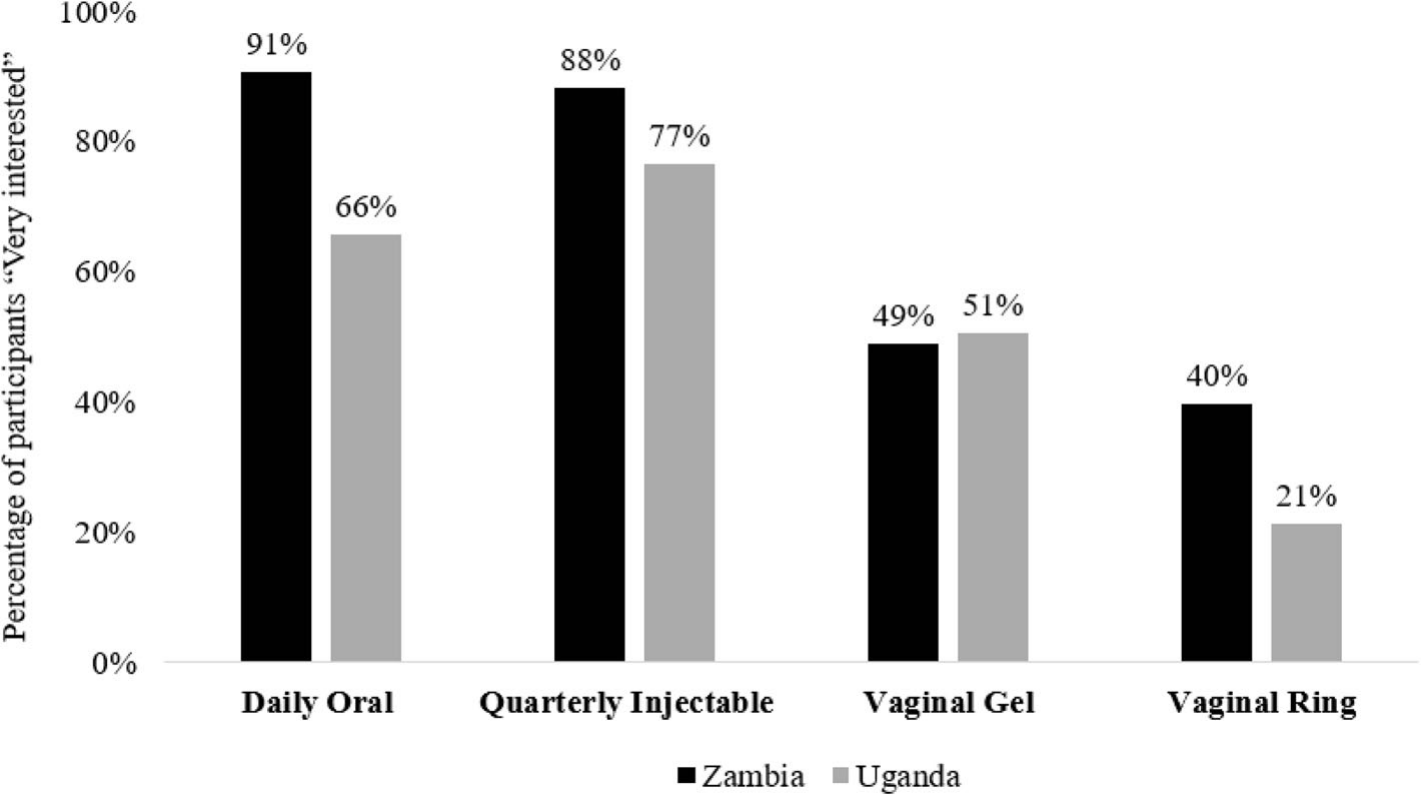
Proportion of participants reporting to be “very interested” in PrEP modality in Zambia and Uganda

PrEP acceptability was high among participants in both studies. Figure 2 shows the percentage of participants that reported being “very interested” in the different PrEP modalities. Almost all participants in Zambia and the majority of participants in Uganda reported being “very interested” in daily oral PrEP. The vast majority of participants in both settings also reported being “very interested” in quarterly injectable PrEP. Interest in vaginally applied PrEP was less common. Roughly half of participants in both settings said they would be “very interested” in PrEP as a vaginal gel, and only 40% of participants in Zambia and 21% of participants in Uganda said they would be “very interested” in PrEP as a vaginal ring.

Almost all participants in both study sites (99% Zambia; 97% Uganda) reported that they would be willing to test for HIV every three months while taking PrEP. The percentage of participants that reported willingness to take PrEP by randomization arm and study site is shown in Table 2. The different HIV self-testing delivery models did not significantly affect participants’ willingness to test for HIV every three months while on PrEP in either Zambia (*P* = 0.64) or Uganda (*P* = 0.19).

**Table 2:** Willingness to be tested for HIV every three months while taking PrEP by randomization arm

	Direct HIVST distribution	HIVST coupon distribution	Standard testing	*P*-value^1^
Zambia	217 (98.2%)	214 (98.6%)	191 (99.0%)	0.64
Uganda	232 (97.1%)	235 (94.4%)	257 (96.5%)	0.19

^1^Estimated using mixed effects logistic regression models with a fixed effect for study arm and a random effect for peer educator group

**Fig. 3:**
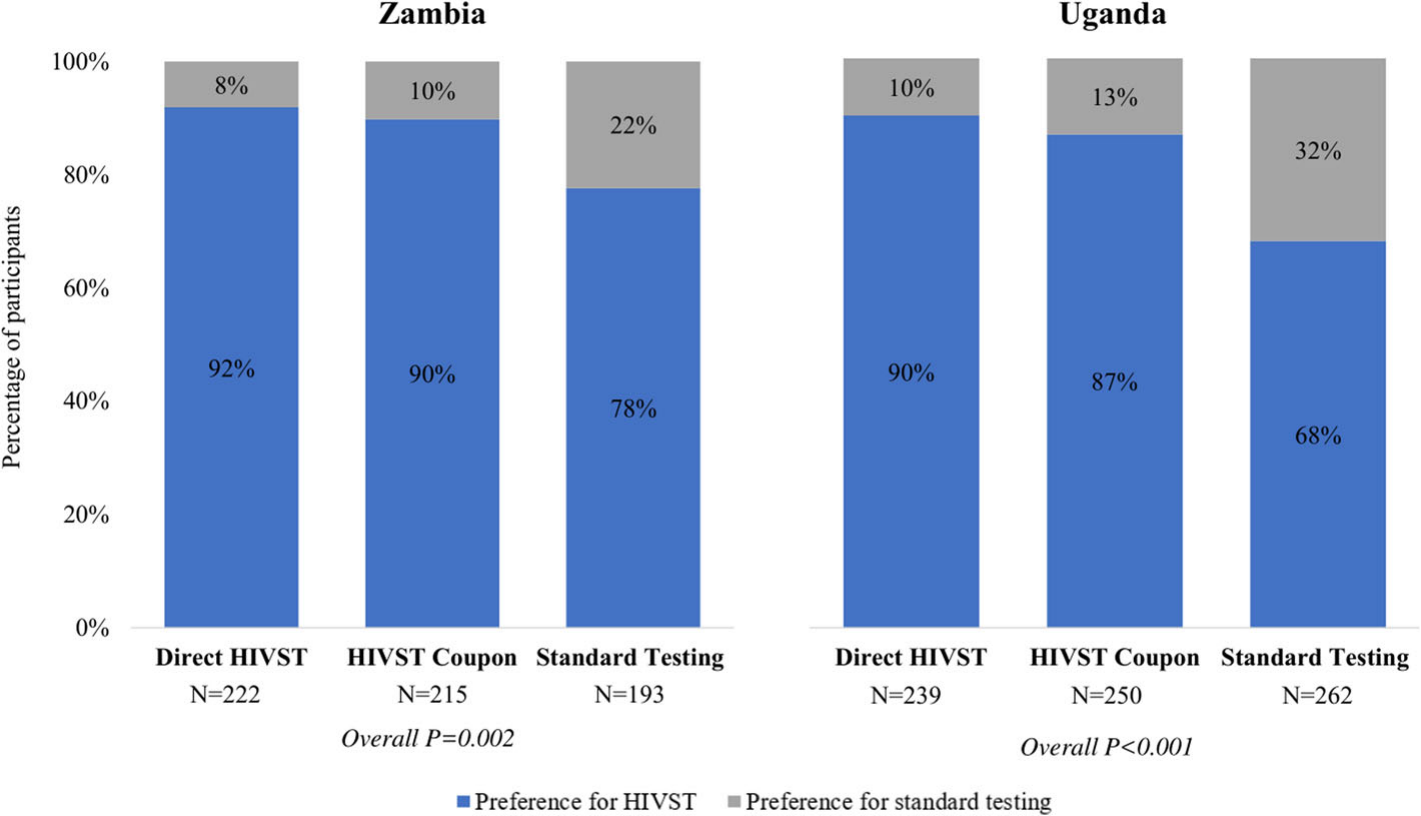
Percentage of participants with a preference for HIV self-testing (HIVST) or standard HIV testing at a clinic while taking PrEP by study randomization in Zambia and Uganda

The different HIV self-testing delivery models did, however, significantly affect participants’ preference for HIV self-testing over standard HIV testing services in both Zambia and Uganda. Figure 3 shows the percentage of participants by randomization arm and study site that reported a preference for HIV self-testing or standard HIV testing services at a clinic while taking PrEP. In both Zambia and Uganda, the percentage of participants that reported a preference for HIV self-testing versus standard HIV testing services while on PrEP was greater in both the HIV self-testing intervention arms compared to the standard of care arm, and differences in this outcomes across randomization arms were statistically significant both in settings (*P* = 0.002 Zambia; *P* < 0.001 Uganda). The majority of all participants both Zambia (87%) and Uganda (81%), however, preferred HIV self-testing versus standard HIV testing services while on PrEP.

## Discussion

FSW in both Zambian transit towns and urban Uganda reported high interest in PrEP and preferred HIV self-testing over standard HIV testing services at clinics to test for HIV regularly (every 3 months) while on PrEP. HIV self-testing for regular, repeat testing was acceptable among FSWs in this study, especially among those who previously had the opportunity to self-test. FSW who had the opportunity to self-test previously may have preferred this approach over standard HIV testing services because they were less intimidated by the new testing technology and had a favorable experience HIV self-testing. These results indicate that HIV self-testing could be used to support PrEP delivery by moving some of the burden of HIV testing outside of health clinics, preventing stigma and discrimination of FSW from healthcare providers and maintaining the confidentiality of FSW taking PrEP [22].

Daily oral PrEP has been shown to be acceptable [29] and implementation to be feasible [20] among other populations of FSWs. In the current study, acceptability of PrEP for prevention of HIV acquisition was high among FSWs in both Zambia and Uganda, but some PrEP modalities were more popular than others. In both study settings, FSWs preferred daily oral PrEP or quarterly injectable PrEP over PrEP in the form of a vaginal gel or ring. This preference is likely explained by the prevalence of various types of non-barrier contraceptive methods available to women in these countries. A recent study conducted among the same population of FSW in Zambia found that the majority of FSW who used a method of non-barrier contraception used injectable contraception; the second most common form of non-barrier contraception was the oral birth control pill [30]. Less than 1 % of Zambian FSW in that study reported use of the vaginal ring [30]. Lack of familiarity with contraceptives inserted in the vagina might have made FSW less interested in PrEP modalities that utilized this strategy.

Acceptability of HIV self-testing was extremely high across randomization arms in both Zambia and Uganda. More than two thirds of participants in the standard of care arm preferred HIV self-testing to standard HIV testing services while taking PrEP in both settings, despite never having had the opportunity to self-test themselves. Willingness to test for HIV every three months was also near universal in both settings, thus suggesting that barriers to HIV self-testing among members of this population are likely attributable to logistics or stigma and discrimination from healthcare providers rather than a lack of will. As demonstrated in the two parent trials, HIV self-testing has the potential to help FSW overcome some of these barriers and achieve both high HIV testing coverage and high levels of repeat testing [22, 26]. A recent study, however, suggests that FSW might have difficulty interpreting HIV self-test results [31], indicating that appropriate pre-test training and on demand support should be considered with the implementation of HIV self-testing.

This study has both strengths and limitations. We found consistent results in two diverse populations of FSWs, improving the generalizability of our findings. Participants were engaged in a randomized trial of HIV self-testing, and thus we were able to compare preferences for HIV self-testing between women who had been randomized to receive a self-test compared to those who did not receive a self-test. Questions related to acceptability of HIV self-testing were therefore not hypothetical for women who had previously had exposure to an HIV self-test. None of the participants in this study, however, actually used or had access to PrEP, and thus they might not have fully understood the research assistants’ explanation of this HIV prevention intervention. Also, as a hypothetical scenario, participants may be more or less likely to indicate that they would use an intervention than they would if they were actually making a decision in real life. Actual behaviors may therefore differ from anticipated behavior. Finally, our selection of study participants may have induced bias in the effect estimates because we selected on a variable (i.e., HIV-negative status) that was reported after randomization. However, PrEP is only indicated for individuals who test negative for HIV, and participants who are living with HIV may have substantially different responses to hypothetical questions regarding HIV prevention. Furthermore, to induce bias, the study randomization arm would have to causally affect HIV status. It is unlikely for this to occur in the one-month timeframe of the study.

## Conclusions

In many sub-Saharan African countries, FSW are a priority population for HIV prevention interventions because of their increased risk of both HIV acquisition and transmission [16, 32]. As more sub-Saharan African countries, including Kenya, Uganda, and South Africa, begin to scale PrEP nationally, understanding preferences for PrEP and interventions for ongoing engagement in care is of increased importance. Here, we found that PrEP is highly acceptable among members of this population and that HIV self-testing is preferred over standard HIV testing services to support regular HIV testing while on PrEP. Previous studies have found HIV self-testing to be effective at increasing frequent testing among FSWs [25, 26]. Policy makers should consider both scaling PrEP to FSW living in high prevalence areas and using HIV self-tests to support PrEP delivery and facilitate detection of breakthrough HIV infections.

## References

[1] Grant RM, Lama JR, Anderson PL, McMahan V, Liu AY, Vargas L (2010). Preexposure chemoprophylaxis for HIV prevention in men who have sex with men. N Engl J Med.

[2] Baeten JM, Donnell D, Ndase P, Mugo NR, Campbell JD, Wangisi J (2012). Antiretroviral prophylaxis for HIV prevention in heterosexual men and women. N Engl J Med.

[3] Thigpen MC, Kebaabetswe PM, Paxton LA, Smith DK, Rose CE, Segolodi TM (2012). Antiretroviral Preexposure prophylaxis for heterosexual HIV transmission in Botswana. N Engl J Med.

[4] Montgomery MC, Oldenburg CE, Nunn AS, Mena L, Anderson P, Liegler T (2016). Adherence to pre-exposure prophylaxis for HIV prevention in a clinical setting. PLoS One.

[5] Volk J, Marcus JL, Phengrasamy T, Blechinger D, Nguyen DP, Follansbee S (2015). No new HIV infections with increasing use of HIV preexposure prophylaxis in a clinical practice setting. Clinical infectious diseases.

[6] Thaden JT, Gandhi M, Okochi H, Hurt CB, McKellar MS (2018). Seroconversion on preexposure prophylaxis. AIDS.

[7] Hurt CB, Eron JJ, Cohen MS (2011). Pre-exposure prophylaxis and antiretroviral resistance: HIV prevention at a cost?. Clin Infect Dis.

[8] WHO (2016). Consolidated guidelines on the use of antiretroviral drugs for treating and preventing HIV infection.

[9] Centers for Disease Control and Prevention (CDC) (2014). Preexposure prophylaxis for the prevention of HIV infection in the United States, 2014: A clinical practice guideline.

[10] Mack N, Odhiambo J, Wong CM, Agot K (2014). Barriers and facilitators to pre-exposure prophylaxis (PrEP) eligibility screening and ongoing HIV testing among target populations in Bondo and Rarieda, Kenya: results of a consultation with community stakeholders. BMC Health Serv Res.

[11] Ngure K, Heffron R, Mugo N, Thomson KA, Irungu E, Njuguna N (2017). Feasibility and acceptability of HIV self-testing among pre-exposure prophylaxis users in Kenya. J Int AIDS Soc.

[12] Figueroa C, Johnson C, Ford N, Sands A, Dalal S, Meurant R (2018). Reliability of HIV rapid diagnostic tests for self-testing compared with testing by health-care workers: a systematic review and meta-analysis. The Lancet HIV.

[13] Figueroa C, Johnson C, Verster A, Baggaley R (2015). Attitudes and acceptability on HIV self-testing among key populations: a literature review. AIDS Behav.

[14] Krause J, Subklew-Sehume F, Kenyon C, Colebunders R (2013). Acceptability of HIV self-testing: a systematic literature review. BMC Public Health.

[15] Stevens DR, Vrana CJ, Dlin RE, Korte JE (2017). A global review of HIV self-testing: themes and implications. AIDS Behav.

[16] Baral S, Beyrer C, Muessig K, Poteat T, Wirtz AL, Decker MR (2012). Burden of HIV among female sex workers in low-income and middle-income countries: a systematic review and meta-analysis. Lancet Infect Dis.

[17] Beyrer C, Crago A-L, Bekker L-G, Butler J, Shannon K, Kerrigan D (2015). An action agenda for HIV and sex workers. Lancet.

[18] Deering KN, Lyons T, Feng CX, Nosyk B, Strathdee SA, Montaner JSG (2013). Client demands for unsafe sex: the socioeconomic risk environment for HIV among street and off-street sex workers. JAIDS Journal of Acquired Immune Deficiency Syndromes.

[19] Cowan FM, Delany-Moretlwe S (2016). Promise and pitfalls of pre-exposure prophylaxis for female sex workers. Curr Opin HIV AIDS.

[20] Eakle R, Gomez GB, Naicker N, Bothma R, Mbogua J, Cabrera Escobar MA (2017). HIV pre-exposure prophylaxis and early antiretroviral treatment among female sex workers in South Africa: results from a prospective observational demonstration project. PLoS Med.

[21] Dudina VI, Bowling JM, King EJ, Dudina VI, Moracco KE (2015). Motivators and barriers to HIV testing among street-based female sex workers in St. Petersburg, Russia. Global Public Health.

[22] Chanda MM, Perez-Brumer AG, Ortblad KF, Mwale M, Chongo S, Kamungoma N (2017). Barriers and facilitators to HIV testing among Zambian female sex Workers in Three Transit Hubs. AIDS Patient Care STDs.

[23] Lancaster KE, Cernigliaro D, Zulliger R, Fleming PF (2016). HIV care and treatment experiences among female sex workers living with HIV in sub-Saharan Africa: a systematic review. Afr J AIDS Res.

[24] Wanyenze RK, Musinguzi G, Kiguli J, Nuwaha F, Mujisha G, Musinguzi J (2017). “When they know that you are a sex worker, you will be the last person to be treated”: perceptions and experiences of female sex workers in accessing HIV services in Uganda. BMC Int Health Hum Rights.

[25] Chanda MM, Ortblad KF, Mwale M, Chongo S, Kanchele C, Kamungoma N (2017). HIV self-testing among female sex workers in Zambia: a cluster randomized controlled trial. PLoS Med.

[26] Ortblad K, Kibuuka Musoke D, Ngabirano T, Nakitende A, Magoola J, Kayiira P (2017). Direct provision versus facility collection of HIV self-tests among female sex workers in Uganda: a cluster-randomized controlled health systems trial. PLoS Med.

[27] Oldenburg CE, Ortblad KF, Chanda M, Mwanda K, Nicodemus W, Sikaundi R (2017). Zambian peer educators for HIV self-testing (ZEST) study: rationale and design of a cluster randomized trial of HIV self-testing among female sex workers in Zambia. BMJ Open.

[28] Zachary D, Mwenge L, Muyoyeta M, Shanaube K, Schaap A, Bond V (2012). Field comparison of OraQuick ADVANCE rapid HIV-1/2 antibody test and two blood-based rapid HIV antibody tests in Zambia. BMC Infect Dis.

[29] Eakle R, Bourne A, Mbogua J, Mutanha N, Rees H (2018). Exploring acceptability of oral PrEP prior to implementation among female sex workers in South Africa. J Int AIDS Soc.

[30] Chanda MM, Ortblad KF, Mwale M, Chongo S, Kanchele C, Kamungoma N (2017). Contraceptive use and unplanned pregnancy among female sex workers in Zambia. Contraception.

[31] Ortblad KF, Musoke DK, Ngabirano T, Nakitende A, Haberer JE, McConnell M (2018). Female sex workers often incorrectly interpret HIV self-test results in Uganda. J Acquir Immune Defic Syndr.

[32] Shannon K, Strathdee SA, Goldenberg SM, Duff P, Mwangi P, Rusakova M (2015). Global epidemiology of HIV among female sex workers: influence of structural determinants. Lancet.

